# A comprehensive study on the efficacy of a wearable sleep aid device featuring closed-loop real-time acoustic stimulation

**DOI:** 10.1038/s41598-023-43975-1

**Published:** 2023-10-16

**Authors:** Anh Nguyen, Galen Pogoncheff, Ban Xuan Dong, Nam Bui, Hoang Truong, Nhat Pham, Linh Nguyen, Hoang Nguyen-Huu, Khue Bui-Diem, Quan Vu-Tran-Thien, Sy Duong-Quy, Sangtae Ha, Tam Vu

**Affiliations:** 1 Department of Computer Science, University of Montana, Missoula, MT 59812 USA; 2 Earable Inc., Boulder, CO 80309 USA; 3 Department of Electrical Engineering, University of Colorado Denver, Denver, CO 80204 USA; 4 Department of Computer Science, University of Colorado Boulder, Boulder, CO 80309 USA; 5 School of Computer Science and Informatics, Cardiff University, Cardiff, CF24 4AG UK; 6 University of Medicine and Pharmacy at Ho Chi Minh City, Ho Chi Minh City, Vietnam; 7 Lam Dong Medical College, Da Lat City, Lam Dong Province Vietnam; 8 Pham Ngoc Thach University of Medicine, Ho Chi Minh City, Vietnam; 9 Hershey Medical Center, Penn State College of Medicine, Hershey, PA 17033 USA; 10 Department of Computer Science, University of Oxford, Oxford, OX1 3QD UK

**Keywords:** Circadian rhythms and sleep, Health care, Electrical and electronic engineering, Mechanical engineering

## Abstract

Difficulty falling asleep is one of the typical insomnia symptoms. However, intervention therapies available nowadays, ranging from pharmaceutical to hi-tech tailored solutions, remain ineffective due to their lack of precise real-time sleep tracking, in-time feedback on the therapies, and an ability to keep people asleep during the night. This paper aims to enhance the efficacy of such an intervention by proposing a novel sleep aid system that can sense multiple physiological signals continuously and simultaneously control auditory stimulation to evoke appropriate brain responses for fast sleep promotion. The system, a lightweight, comfortable, and user-friendly headband, employs a comprehensive set of algorithms and dedicated own-designed audio stimuli. Compared to the gold-standard device in 883 sleep studies on 377 subjects, the proposed system achieves (1) a strong correlation (0.89 ± 0.03) between the physiological signals acquired by ours and those from the gold-standard PSG, (2) an 87.8% agreement on automatic sleep scoring with the consensus scored by sleep technicians, and (3) a successful non-pharmacological real-time stimulation to shorten the duration of sleep falling by 24.1 min. Conclusively, our solution exceeds existing ones in promoting fast falling asleep, tracking sleep state accurately, and achieving high social acceptance through a reliable large-scale evaluation.

## Introduction

Getting enough sleep is essential to health and well-being. However, it has been reported that hundreds of millions globally are getting insufficient high-quality shut-eye caused by sleep disorders^1,2^. Due to associated adverse outcomes^3–5^, improving sleep quality has become a growing trend to incorporate sleep in health promotion and disease prevention^6^. Among several symptoms of insomnia, difficulty falling asleep is the most common trouble, highly ruining the whole night sleep session and increasing sleep debt^2^ and many chronic health conditions^7^. Thus, treatment for such sleeplessness is critical to enhancing the quality of life.

Traditional treatments for trouble falling asleep consist of complementary medicine^8,9^, sleep supplements^10^, and natural remedies^11–13^. Although producing quick symptomatic relief, drugs are associated with side effects and short sustainability^14^. Similarly, natural solutions are limited due to a need for therapists or daytime consumption. Overcoming these limitations introduced Internet-based cognitive behavioral therapy for insomnia (ICBT-i)^15^ given through hi-tech devices right at sleeping time. Studies demonstrated that this therapy has significantly reduced sleep onset latency (SOL), a crucial factor in evaluating the falling asleep difficulty level^16,17^. However, ICBT-i has not been ideal yet because it continues even after users fall asleep, negatively waking them up or keeping their sleep shallow.

The rise of bio-sensing technologies^18–20^ has become a boon for sleep awareness. Beyond medical-grade devices, referred to as polysomnography (PSG)^21^, there have been various hi-tech solutions assessing sleep quality, such as wristbands^22–24^, headbands^25–28^, ear-worn devices^29,30^, contactless sensory systems^31–38^, and mobile apps^39–41^, to name a few. Unlike PSG, these are compact, inexpensive, lightweight, and easily used for multiple nights at home without expert supervision. However, beyond performing sleep monitoring as PSG, only a few consider addressing sleep issues. Additionally, with a limited number of studies, their effectiveness has still been questioned, motivating our development of an advanced bio-wearable for efficaciously assisting sleep improvement.

**Figure 1:**
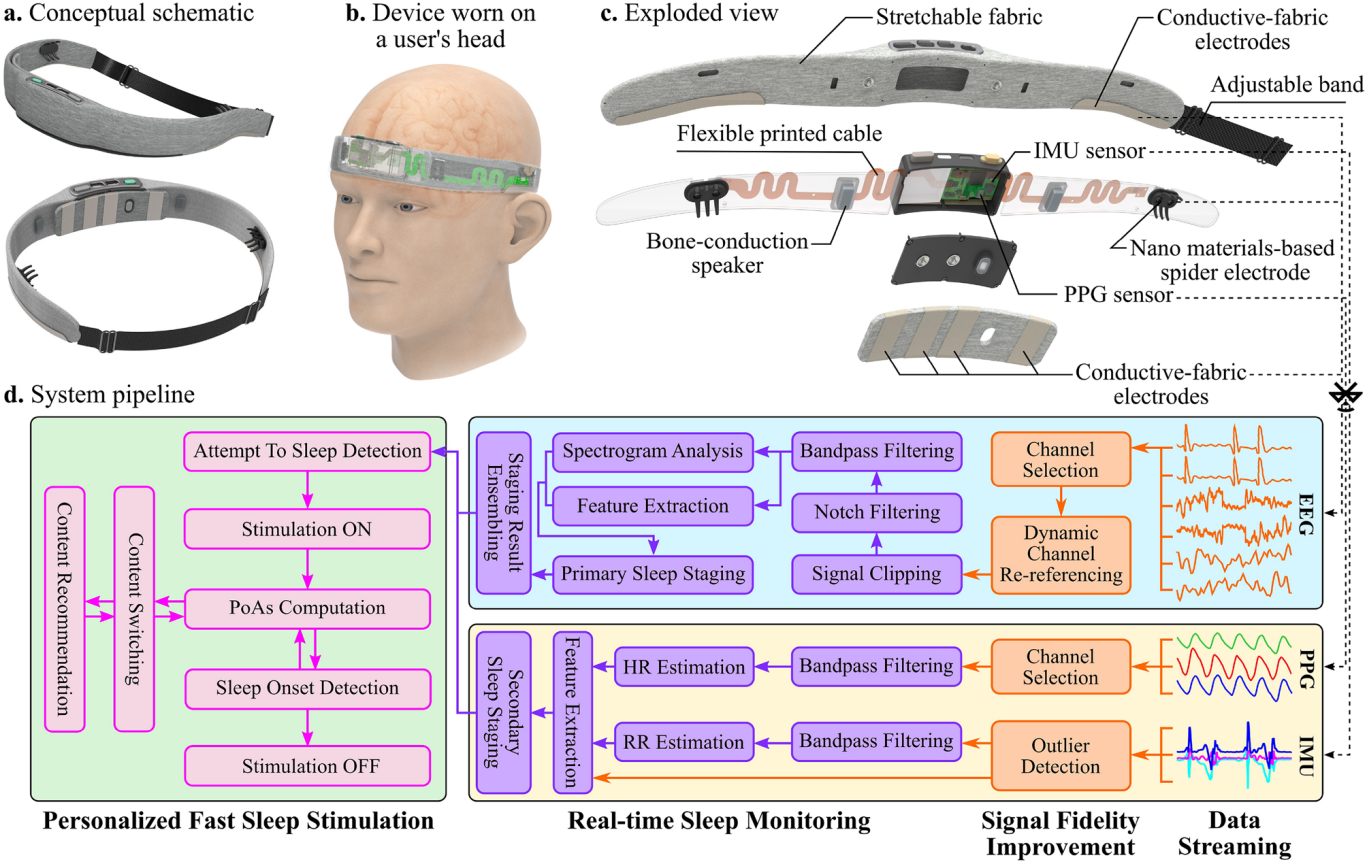
Earable overview. (**a**) Device design. (**b**) Device placement on a user’s head. (**c**) Exploded view. (**d**) End-to-end system pipeline.

In this work, we propose a novel sleep aid solution to meet three goals: highly qualified bio-signal acquisition, accurate real-time sleep monitoring, and effective fast sleep promotion. The contributions of our proposed system are (1) a real-time, closed-loop feedback model to promote faster sleep, (2) a compact and comfortable wearable design to facilitate sensing and stimulating functions, and (3) a large-scale evaluation to prove the effectiveness of our proposed solution. Namely Earable, our system is a headband composed of eight dry biopotential electrodes, a 3-axis accelerometer, a photoplethysmographic (PPG) sensor, and two bone-conduction speakers. Figure 1 presents a concept of our sleep aid framework. However, realizing such a system involves multiple challenges ranging from system design to signal variability and user dependence. Therefore, through multiple unit tests, we first show that Earable owes signal quality comparable to PSG for bio-signal acquisition. Then, we demonstrate its power to robustly track sleep and eliminate a reasonable amount of SOL through auditory stimuli delivery in a comprehensive study conducted on 377 subjects over 883 sleep sessions in various protocols. In conclusion, this paper presents Earable, a compact, accurate, and user-friendly biosensing and feedback system, which supports not only sleep but also many future healthcare and human-computer interaction applications.

## Results

### System implementation

Figure 1 presents our full-stack wireless head-worn system, Earable, incorporating several sensors and dedicated algorithms in a single sleep aid platform. Figure 1a–c illustrates its prototype, on-head placement, and exploded view, respectively, with key designs to achieve high performance and user experience. Firstly, we design and fabricate electrodes in different shapes and materials based on their position to unobtrusively measure low-amplitude bio-signals. Specifically, Earable uses stretchable conductive fabric made of antioxidant silver-coated polyurethane for electrodes on the forehead and above the ears. The other ones positioned on the hairy area are made of flexible conductive silicone with six prongs for maximizing hair penetration and long-lasting wear. We also design a strip detachable from the mainboard for easy forehead electrode sanitization. Secondly, we integrate the accelerometer and PPG sensor to monitor users’ sleep posture, heart rate (HR), and respiratory rate (RR) over time as complementary to strengthen our sleep stage inference algorithm. Thirdly, Earable embeds two bone-conduction speakers for auditory stimulation that helps minimize the air-traveling sound possibly affecting users’ partners while keeping a perceptive loudness. Finally, we connect all components to the mainboard using flexible printed circuits and place the board in a lightweight 3D-printed box to protect them from users’ unconscious movements during sleep. Likewise, we properly shield the speakers’ wires from the mainboard to minimize acoustic artifacts on bio-signals. Accordingly, we embed this hardware into a headband to acquire and stream the bio-signals via Bluetooth to a host device, where we deploy core algorithms (Fig. 1d). These processing modules extract sleep features and infer sleep stages to monitor and promote sleep by automatically controlling the play of auditory stimuli in real-time. We present all algorithms in detail in the Supplementary Materials.

### Assessment of reliable bio-signal acquisition

**Figure 2:**
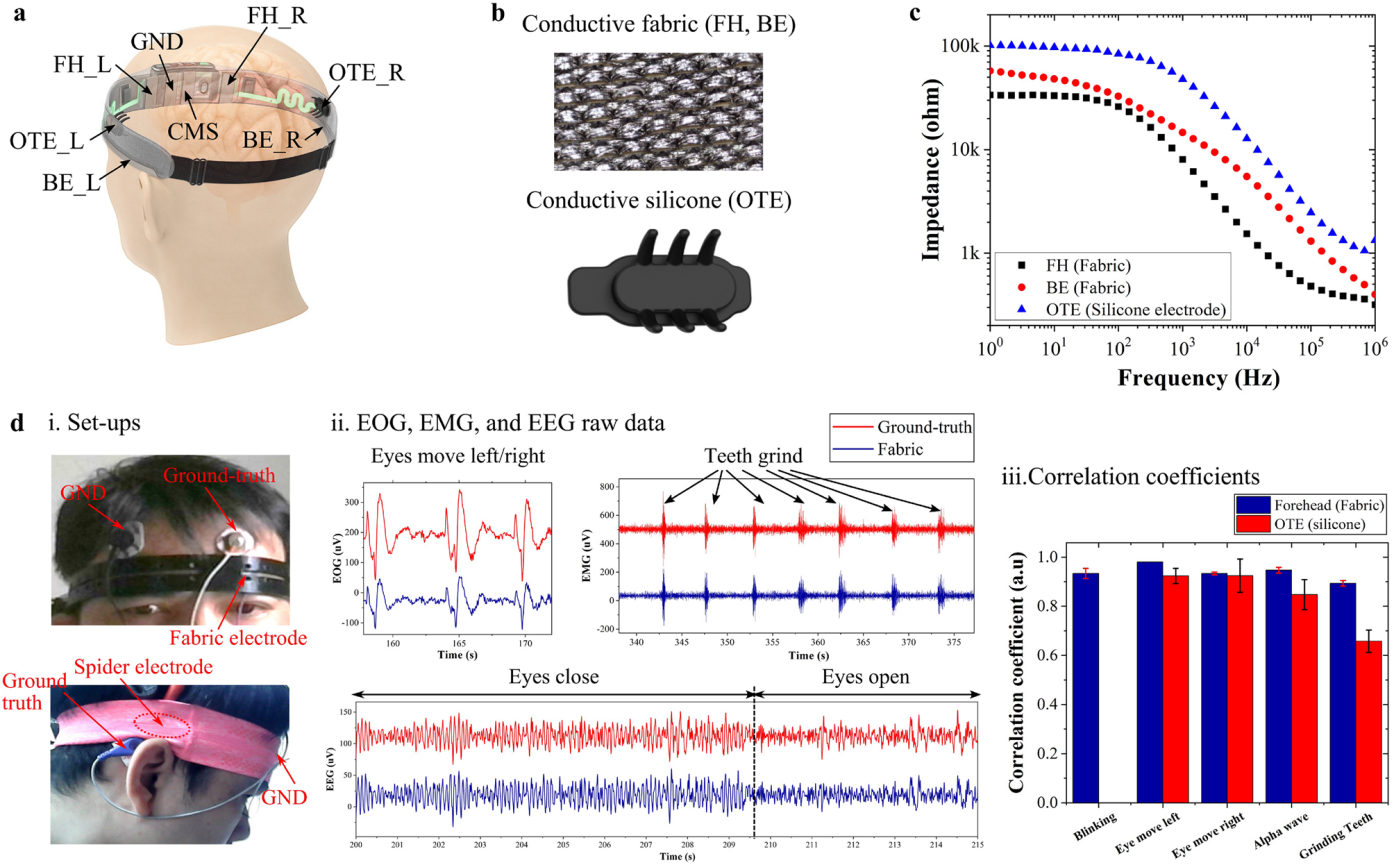
Electrode placement and electrophysiological recording using Earable. (**a**) Name of biopotential electrodes located on the headband. (**b**) Micrograph of fabric and conductive silicone electrodes. The scale bar is 500$\upmu$m. (**c**) Frequency-dependent impedance of FH, BE, and OTE electrodes. (**d**) (i) Experimental setup for biopotential correlation tests for fabric FH (top) and conductive silicone OTE (bottom) electrodes. (ii) Exemplary electrophysiological signals during several bio-calibration events collected from the FH location. Data is shifted vertically for clarity. (iii) Correlation coefficients between electrophysiological signals collected using our dry electrodes and the clinical standard wet Ag/AgCl electrodes at the FH and OTE locations. Note that the eye blinking is not observable at the OTE location.

We position the electrodes symmetrically on the forehead (FH) and behind (BE) and over (OTE) the two ears (Fig. 2a). Figure 2b provides zoom-in images of their conductive fabric and silicone materials with surface resistance of $\sim$1–2$\Omega$.cm$^{-1}$ and $\sim$100–200$\Omega$.cm$^{-1}$, respectively. We first measured the electrode impedance from 1Hz to 1MHz using electrochemical impedance spectroscopy. Figure 2c demonstrates that their impedance is frequency-dependent and increases with reducing frequency. Specifically, the OTE electrode has the highest impedance due to the small contact area at its prongs’ tip. On the other hand, the impedance of the FH electrode is lower than the BE one, even though being made of the same materials. It indicates that the impedance is intimately tight to their location, different in the contact area and/or the bone/skin structure. Besides, all electrodes have very weak frequency-dependent impedance behavior in the 1–100Hz range, demonstrating a high contact quality between them and the skin so that their interface behaves like a pure resistor^42^. Altogether, high conductivity, low impedance, and intimate electrode-skin contact enable high-quality electrophysiological signal acquisition of our electrodes.

Next, we conducted event-related potential (ERP) tests: tracking eye movements (EOG), muscle activity during teeth grinding (EMG), and spontaneous Alpha rhythm during relaxation (EEG). Figure 2d(i) illustrates the FH and OTE electrode hook-ups with ground-truth hydrogel electrodes attached nearby for signal comparison. Figure 2d(ii) shows exemplary EOG, EMG, and EEG signals collected at the FH location. For all events, the signals collected using our electrodes are incredibly similar to those collected by the hydrogel. They can obtain the low-frequency (<4Hz) EOG, the high-frequency ($\sim$100Hz) EMG, and the Alpha (8–13Hz) EEG. We further computed the correlation coefficient between the signals collected from both electrode types to quantify our signal quality. High values in Fig. 2d(iii) confirm that they are strongly correlated, agreeing with the result observed in Fig. 2d(ii). At the FH location, the correlation coefficients are 0.93 ± 0.02, 0.98 ± 0.00, 0.93 ± 0.01, 0.95 ± 0.01, and 0.89 ± 0.01 for eye blinking, eye moving left and right, alpha brainwave, and teeth grinding, respectively. Disregarding eye blinking as the electrode is far away from the eyes, they are 0.92 ± 0.03, 0.92 ± 0.07, 0.85 ± 0.06, and 0.66 ± 0.05, respectively, at the OTE location. These are slightly lower than at the FH location due to its higher impedance shown earlier.

**Figure 3:**
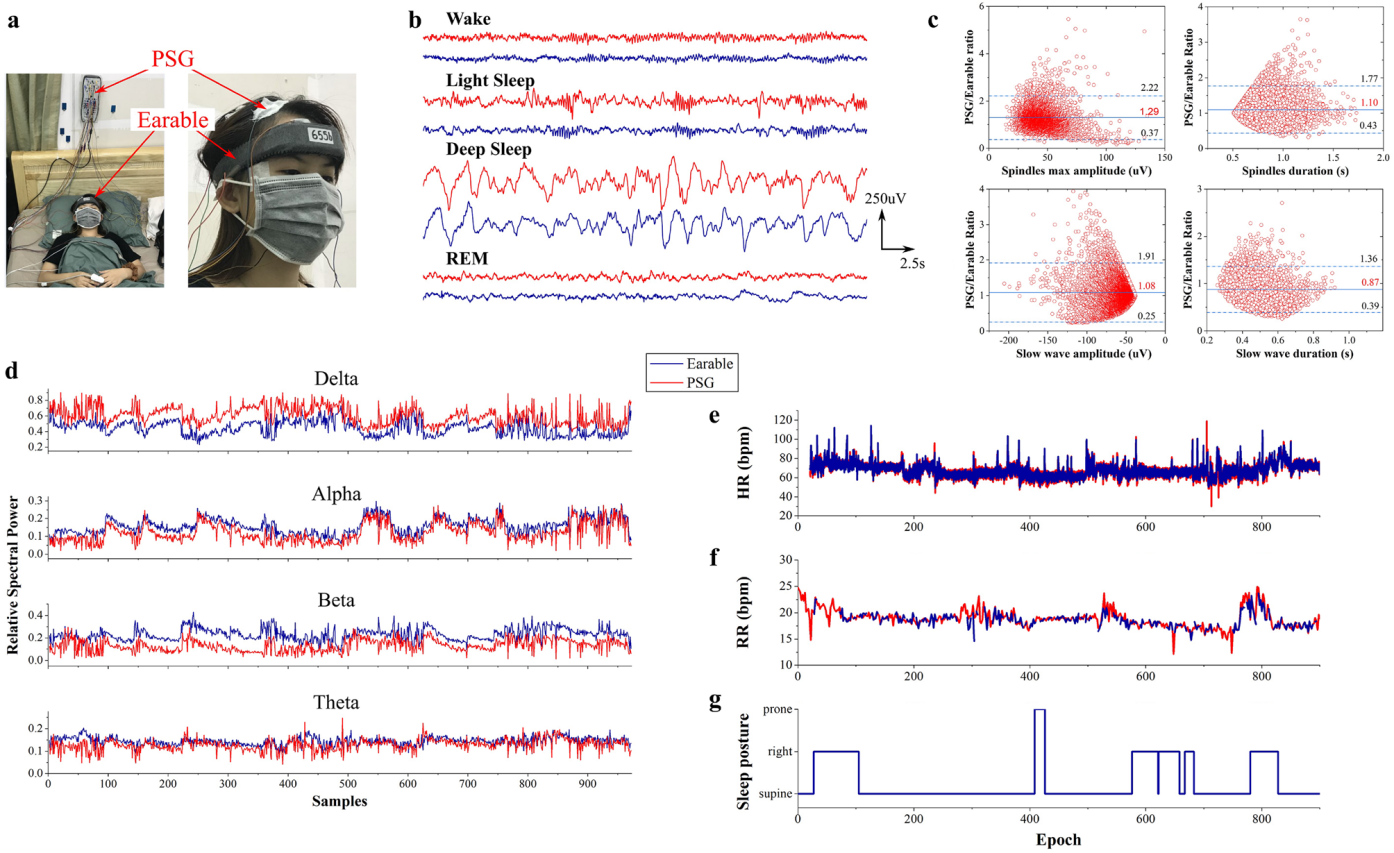
Signal quality using Earable in comparison with PSG in actual sleep studies. (**a**) Clinical setup. A participant wears our headband and is hooked up to the PSG device at the same time for simultaneous validation of sleep microstructures and other vital signs. Note that wearing a face mask, which is not part of our system, is to comply with the CDC’s guidelines issued during the Covid-19 pandemic only. (**b**) Comparison of raw electrophysiological signals collected by two systems at four different sleep stages. (**c**) Bland-Altman analysis of slow waves and spindles. (**d**) Relative spectra power of Delta, Theta, Alpha, and Beta brainwaves collected during sleep. (**e,f**) Comparison of heart rate (**e**) and respiratory rate (**f**). (**g**) An example of a user’s sleep posture throughout one full-night sleep.

Having demonstrated the high fidelity of signals, we further studied the electrodes’ efficacy in overnight sleep sessions. Figure 3a depicts a participant wearing Earable and hooked up to the PSG at eight channels, including F3/A2, F4/A1, C3/A2, C4/A1, O1/A2, O2/A1, LOC/A2, and ROC/A1. Figure 3b displays 8-second signal samples recorded simultaneously at Earable’s FH_L and PSG’s F3/A2. Consistent with prior results, our signals are remarkably similar to those collected from PSG in all four sleep stages. Therefore, our system can capture sleep hallmarks traditionally observed in sleep studies, including Alpha brainwaves in the wake stage (W), spindles and K-complex in light sleep (LS), and Delta waves in deep sleep (DS)^43^.

We also quantified the data quality by performing the Bland–Altman analysis^44^ on the sleep hallmarks: slow waves’ duration and negative amplitude and spindles’ frequency and max amplitude. Figure 3c compares them by plotting their mean value against the ratio between the measurement values. For each characteristic, we show the mean difference between the two paired measurements (solid blue) and 95% limits of agreement (dashed blue). Overall, we found that sleep hallmarks measured by both systems were equivalent. Note that our values are not so high as those reported in Leach et al.’s work^44^ because our electrode placement is not the same as in the PSG montage, resulting in amplitude differences. We found that, on average, the amplitude of spindles and slow waves measured by PSG was about 29% and 8% higher, respectively, than Earable. However, as shown in the following sections, these differences do not affect our signals’ high fidelity and the system’s sleep-scoring capability.

In Fig. 3d, we computed the relative spectral power (RSP) relevant to specific brainwaves. Although not strictly equal, the data from Earable and PSG followed the same trend through different sleep stages. To estimate the RSP agreement between two systems, we further computed the mean absolute error (MAE) and the Pearson correlation (PC) at each frequency band. For Delta, Theta, Alpha, and Beta bands, the MAE values are 0.1636, 0.0213, 0.0402, and 0.1013, and the PC values are 0.7030, 0.5026, 0.8794, and 0.4877, respectively. These indicate their good agreement throughout the full-night sleep session.

Besides, Earable collects HR, RR, and sleep postures (SP) to demonstrate its full capability in sleep-related data description. Specifically, our system extracted HR from the PPG data (Supplementary Func. SF 1) and RR (Supplementary Func. SF 2) and SP from the IMU values. Figure 3e,f exhibit that the HR and RR extracted from Earable follow almost precisely the ground-truth data from a pulse oximeter and a breathing belt connected to PSG. Particularly, our HR measurement shows an MAE of 2.19 ± 2.84 bpm, accompanied by a Mean Absolute Percentage Error (MAPE) of 3.56%. Our RR calculation exhibits an MAE of 0.96 ± 1.61 bpm with a MAPE of 6.09%. Finally, Fig. 3g shows that our device can infer subjects’ SP.

### Real-time sleep scoring performance in overnight sleep studies

**Figure 4:**
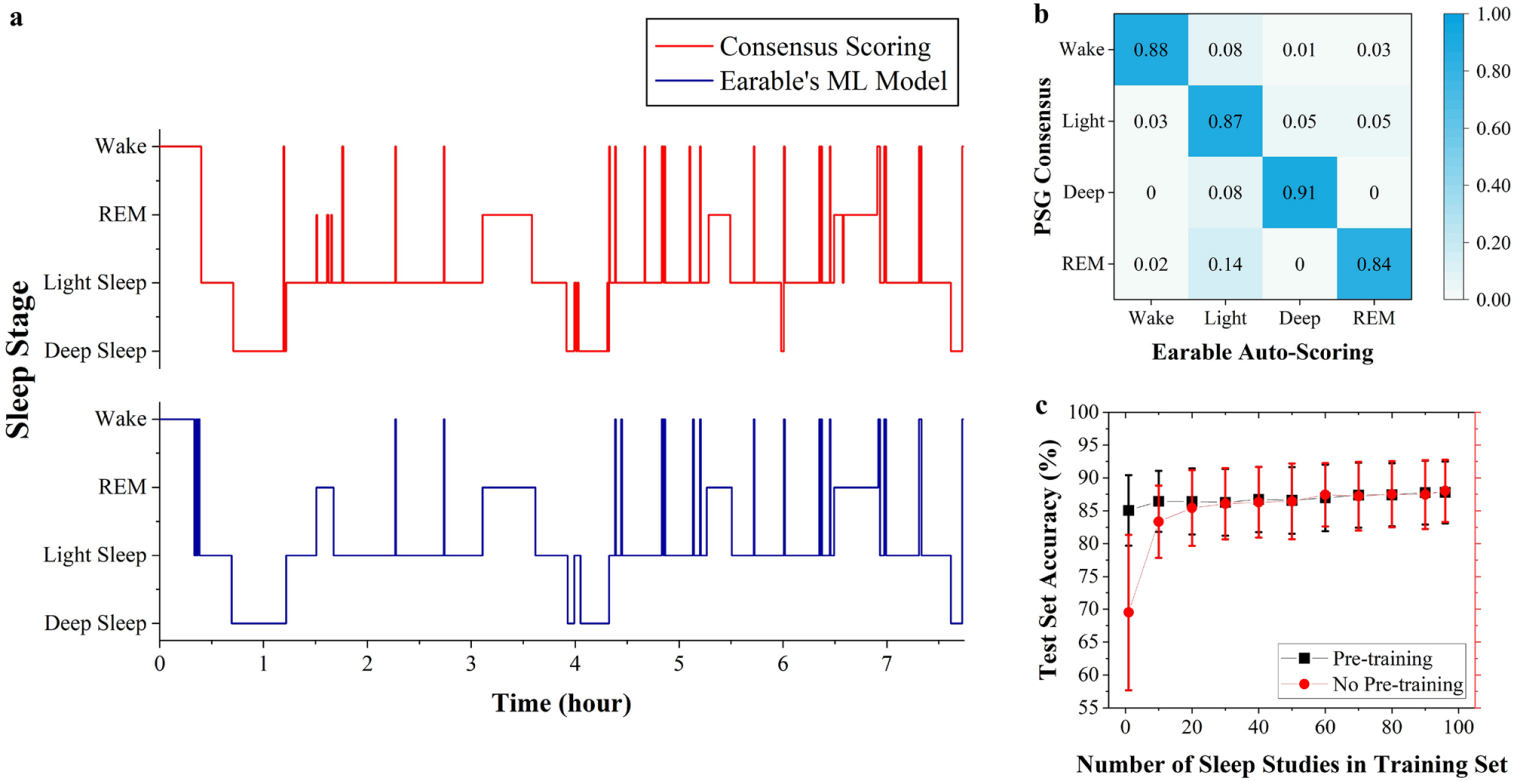
Accuracy performance of the Earable sleep stage classification model in overnight sleep studies. (**a**) Comparison of hypnograms inferred from the Earable model and scored by a sleep technician in one study. (**b**) Confusion matrix. (**c**) Impact of training dataset size on the PML model performance.

Figure 4a depicts 4-stage hypnograms scored by a trained sleep technician and the Earable sleep scoring algorithm. Given the imbalanced distribution of sleep stages in typical nocturnal sleep^45^, we evaluate precision, recall, and F1-score metrics for each of them. We then use multi-class accuracy to summarize the agreement between our algorithm output and the consensus scoring. We utilized rigorous k-fold cross-validation and independent testing to evaluate model performance. The original 155 sleep studies were partitioned into 106 for cross-validation and 49 for final independent testing, enabling robust model development. Specifically, our model demonstrated an accuracy of 84.08 ± 1.42% averaged over 11 folds. The comprehensive model development process and performance are detailed in Supplementary Mod. SM 4 and Supplementary Table ST 1. The model was finalized based on k-fold insights before evaluating the independent test set of 49 studies. This strict separation between k-fold cross-validation and independent testing enabled an unbiased assessment of model performance. As averaged over the independent test set, we achieved an accuracy of 87.8 ± 5.3%. Supplementary Tab. ST 2 provides F1-score, precision, and recall values achieved for individual sleep stage. In Fig. 4b, we provide a confusion matrix summarizing model behavior on this test set. To avoid the inevitable variability and disagreement between different ways of sleep scoring, we computed and achieved Cohen’s kappa coefficient^46^ of 0.83 as a strong agreement^47^. For comparison, the Cohen’s kappa coefficient between the manual scorings done by each sleep technician and their consensus was 0.92 on average.

On the other hand, developing a generalizable sleep scoring model regardless of inter-user signal variability usually requires sleep data from a wide user population. Hence, Fig. 4c illustrates model performance as a function of training set size when using the primary machine learning (PML) model with and without pre-trained weights. When the training data is limited ($\le$20 sleep studies), the influence of pre-training becomes significant in mitigating the PML model overfitting. As more training studies become available, we observe similar test-set performance between PML with pre-trained and randomly initialized weights. Moreover, the accuracy increases when the training set keeps growing but reaches a point where the average accuracy among all test-set studies stops increasing with statistical significance. The arrival at such a plateau indicates a sufficient training dataset size is available, given the PML model architecture and the test dataset.

### Efficacy of channel selection and significance of dynamic re-referencing scheme

**Figure 5:**
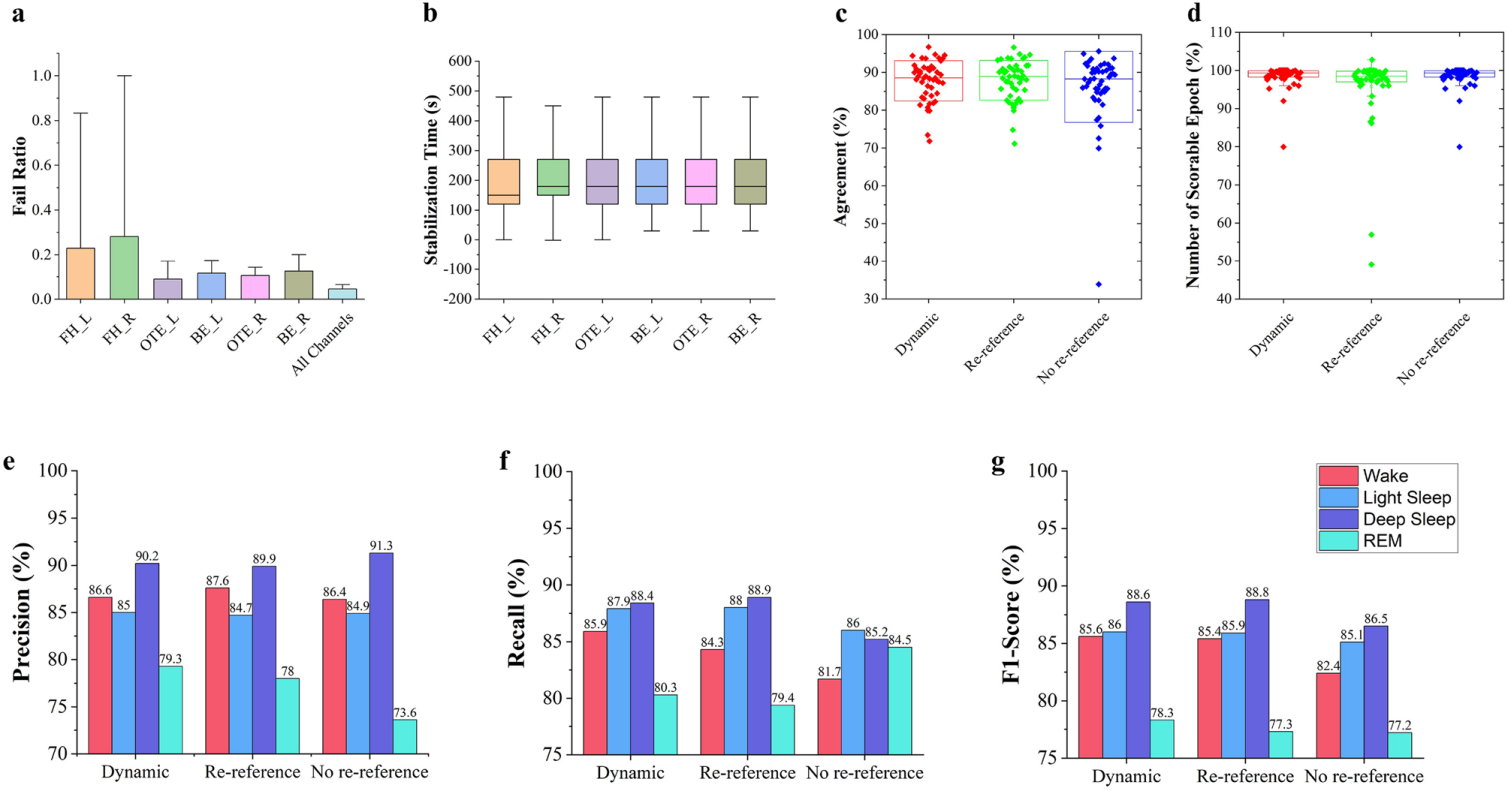
Accuracy performance of the Earable sleep scoring model validated in modules. (**a**,**b**) Signal quality validation for individual channels in terms of fail ratio (**a**) and stabilization time (**b**). (**c**–**g**) Accuracy performance in various channel re-referencing schemes.

Electrode-skin contact quality is highly disturbed by users’ movements in at-home use cases. Consequently, pre-specified channels are unlikely to be consistently available for sleep staging. Therefore, we develop a Channel Selection module (Supplementary Mod. SM 1) to identify channels containing a high enough fidelity signal for sleep staging. Figure 5a depicts the proportion of epochs the model rejects from each electrode. Evaluating the 49-subject test set, we observe that the OTE_L channel has the greatest availability with a minimal fail rate of 4.59%, followed by OTE_R (6.55%), BE_L (7.30%), BE_R (8.22%), FH_L (19.12%), and FH_R (24.96%). Especially, our data was unscorable only 1.58% of the time when considering all channels together. Reviewing recorded videos, the most common reason for channel unavailability was the headband movement, resulting in improper contact. Sometimes, it caused long-term channel unavailability. Otherwise, the signal could quickly re-stabilize due to the highly conformal electrode materials (Fig. 5b). Note that contradicting their lowest impedance (Fig. 2c), FH channels possess the highest failed rates because the current design makes them more prone to head movements, which can be improved by redesigning the headband for better movement tolerance.

- Scheme 1: Dynamic re-referencing (Supplementary Mod. SM 2)
- Scheme 2: Re-referencing, where each channel is re-referenced to the BE channel on the opposite side
- Scheme 3: No re-referencing, where all channels are recorded using their standard reference

Figure 5c summarizes the distribution of scoring agreement over the 49-subject test set in each scheme. We reached the greatest agreement by always re-referencing the channels to the appropriate BE. However, a one-way ANOVA (Analysis of Variance) test reveals that the mean accuracy across schemes are not significantly different at the 0.05 significance level (p* = 0.388). In Fig. 5d, we observe a difference in the percent of scorable epochs (i.e., epochs with at least one scorable channel) between these schemes (i.e., 98.42%, 96.47%, and 98.42% using Scheme 1–3, respectively). Scheme 2 is the least robust to noisy signals. Even though Scheme 1 and 3 enabled a greater scorable epochs percentage, posthoc tests show that these differences are not statistically significant (p* = 0.068). Figure 5e–g further depict their per-stage precision, recall, and F1-scores. Although having the same statistical mean accuracy, Scheme 1 overperforms the others regarding flexibility.

### Scoring in the absence of high-fidelity electrophysiological signals

As Earable needs real-time scoring to control the closed-loop stimulation, we utilize other sensing modalities to substitute unscorable epochs. We build a secondary machine learning (SML) algorithm leveraging HR, RR, and SP derived from the PPG and IMU sensors for scoring. Likewise, we employed k-fold cross-validation, yielding SML’s average accuracy of 60.72 ± 7.68% over 14 folds. Detailed information regarding SML’s development, training, and validation can be found in Supplementary Mod. SM 5, while the results derived from k-fold cross-validation are presented in Supplementary Tab. ST 3. Beyond that, we develop an offline rule-based smoothing model (Supplementary Mod. SM 6) to further reduce the number of unscorable epochs for a historical hypnogram review after sleep. To understand the sleep scoring performance using PML, SML, and offline models, we conducted the evaluation using nine sleep sessions under five cases.

- Case 1: Scoring with SML model only (ignoring epochs with excessively noisy PPG or IMU data)
- Case 2: Scoring with PML model only (ignoring epochs left unscored by PML model)
- Case 3: Scoring with PML and offline models
- Case 4: Scoring with PML and SML models
- Case 5: Scoring with PML, SML, and offline models

**Figure 6:**
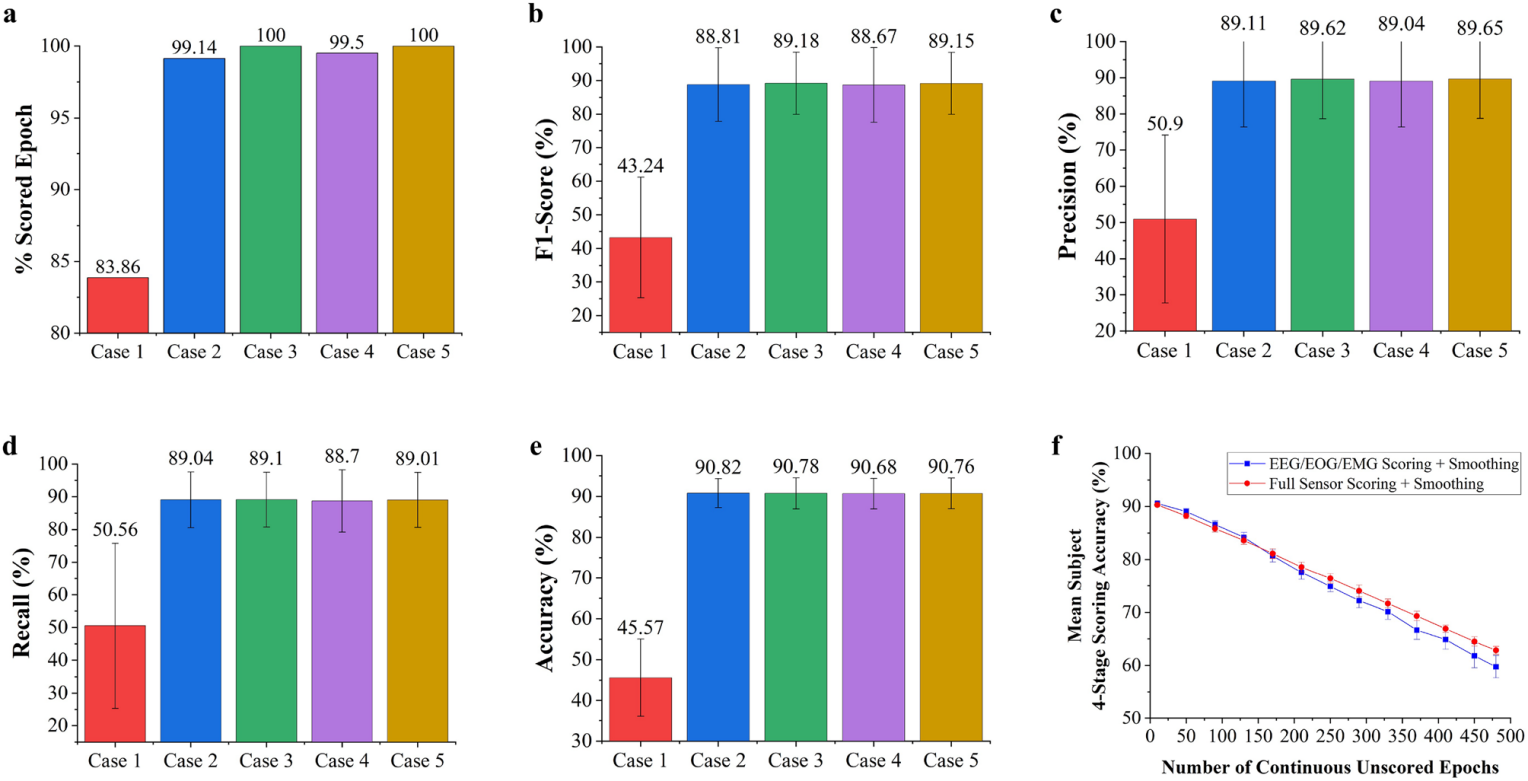
Further improvement of sleep scoring via smoothing and full sensor. (**a**–**e**) Case 1: Scoring with IMU and PPG only. Case 2: Scoring using only ExG (including EEG, EOG, and EMG) data with no smoothing. Case 3: Scoring using only ExG data with HMM smoothing. Case 4: Scoring using all sensors with no smoothing. Case 5: Scoring using all sensors with HMM smoothing. (**f**) Scoring in the Presence of Long Poor Quality EEG Signals.

Figure 6a demonstrates that the SML and offline models enable more scorable epochs than when applying the PML model alone (permitting 100% of epochs, including originally unscored ones, to be scored). Figure 6b–e show F1-score, precision, recall, and accuracy values, respectively, under the five cases. We observe the accuracy of the SML model only is at 45.57% (Fig. 6e) with per-class F1-scores of 41.66% (W), 37.71% (LS), 59.84% (DS), and 47.20% (REM). In a one-way ANOVA test, we observe no statistically significant difference in the accuracy, macro-precision, macro-recall, or macro-F1 between Case 2–5 (p = 0.9999, p = 0.9979. p = 0.9989, and p = 0.9963, respectively). In Case 3 and 5, however, our proposed system scores every epoch in the test dataset, demonstrating the retention of accuracy after scoring originally ignored epochs. It especially helps remove unlikely sleep stage transitions inferred by PML and SML models when processing abnormal signals. The statistically similar accuracies between Case 3 and 5 may presume the SML model is unnecessary in the processing pipeline. However, removing this model will limit the offline smoothing algorithm because inference becomes less accurate as the duration of an unscorable segment increases, resulting from less local context for rule-based decisions and a decreasingly confident path in the Viterbi algorithm. We demonstrate this behavior by performing an experiment where segments of increasing duration were synthetically removed from the estimated hypnograms of the test set. Specifically, we first remove contiguous segments ranging from 5 min to 4 h randomly selected in each of the estimated hypnograms. Next, in one scenario, we apply the SML model to score these missing epochs using the IMU and PPG data, while in an alternate scenario, we leave these unscored epochs as is. We finally apply the offline smoothing algorithm in both scenarios to score any remaining unscored epochs. This process accuracy was averaged over 50 trials to reduce the variability in performance. As apparent in Fig. 6f, the SML model becomes advantageous to offline scoring performance if the duration of electrophysiological unavailability is >75 min.

### Sleep faster program performance via real-time closed-loop audio stimulation

**Figure 7:**
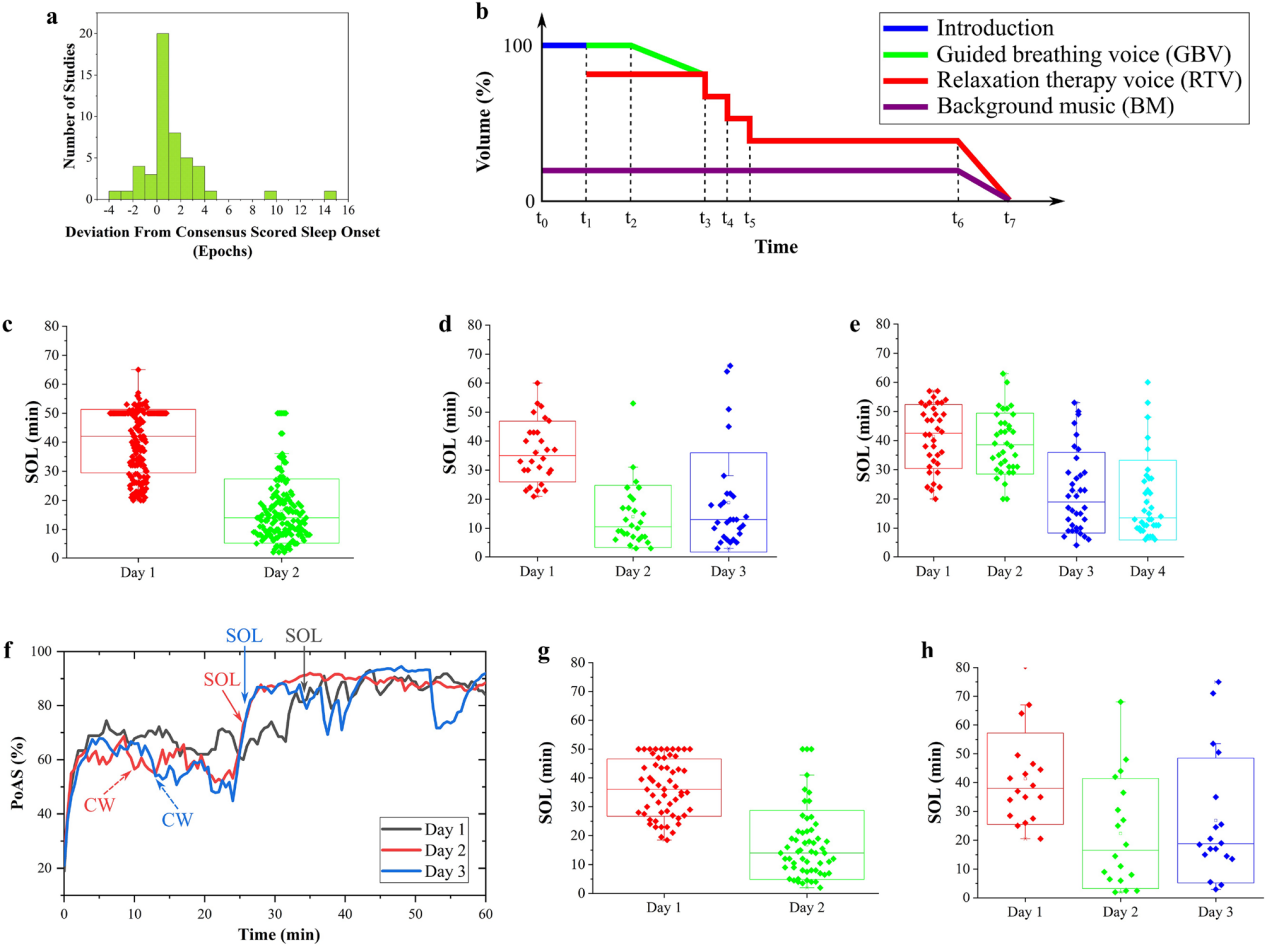
Performance of our proposed sleep faster program. (**a**) Statistical distribution of deviation of sleep onset latency (SOL) estimation between Earable and PSG. (**b**) The structure of stimulation audio/voice used in our stimulation protocols. (**c**) SOL results of the 2-day nap protocol using PSG. Stimulation is used on Day 2 and not used on Day 1. (**d**) SOL results of a 3-day nap protocol using PSG. Stimulation is used on Days 2, 3 and not used on Day 1. (**e**) SOL results of the 4-day nap protocol using PSG. Stimulation is used on Days 3, 4 and not used on Days 1, 2. (**f**) Example of Probability of Being Asleep (PoAs) of a user at the beginning of 3 sleep sessions Day 1, 2, and 3. Closed-loop stimulation is used on Days 2 and 3. The solid arrows indicate the SOL measured by sleep staging. The dashed arrows indicate the occurrence of content switching (CW) during the closed-loop stimulation on Days 2 and 3. (**g**) SOL results of a 2-day nap protocol using Earable. Stimulation is used on Day 2 and not used on Day 1. (**h**) SOL results of 3-day full night sleep protocol using Earable. Stimulation is used on Days 2 and 3 and not used on Day 1.

Sleep onset latency (SOL) is defined as the time when a person undergoes the transition from wake to other sleep stages, usually in the 10–20-min range for a healthy adult^45^. In this section, we demonstrate Earable can accurately detect SOL and reduce the SOL of participants experiencing difficulty falling asleep (SOL > 20 min) via real-time closed-loop audio stimulation. First, in Fig. 7a, we show the deviation of SOL measured by our system from PSG data. The result shows that 96% of them have SOL estimated by Earable within ±5 epochs (2.5 min) of the one extracted from PSG, indicating the high accuracy of our algorithm in SOL detection.

Next, we use auditory stimuli built using the structure shown in Fig. 7b to produce the best stimulation. We divided the evaluation of stimulation influence on SOL into two phases. First, we ran nap sessions on two consecutive days: participants did not listen to any on Day 1 and were subjected to the stimulation on Day 2. During this phase, we used PSG to capture their SOL, played the auditory stimulus through the Earable two bone conductors, and turned it off after 50 min regardless of their sleep status. Figure 7c shows the results of this PSG 2-day nap protocol. We successfully achieve a great SOL reduction from the participants when applying the audio stimulation. After testing on 166 participants, Earable can reduce the SOL from 40.3 ± 11.0 to 16.2 ± 11.1 min on average. To prove the SOL reduction lasts consistently across uses, we further conduct the PSG 3-day nap protocol. This experiment had no audio stimulation on Day 1 but not on Days 2 and 3. Similarly, Fig. 7d demonstrates that the average SOL over 28 participants is 36.4 ± 10.5, 14±10.7, and 18.8 ± 17.2 min for Days 1–3, respectively. We then ran the PSG 4-day nap protocol (no audio stimulation on Days 1–2 and introduction of audio stimulation on Days 3–4) over 36 participants to prove that the “first-time” effect does not cause the SOL reduction. Figure 7e reveals that the average SOLs are 41.2 ± 11.0, 38.9 ± 10.5, 22.1 ± 13.9, and 19.5 ± 13.4 min, respectively.

Finally, in the second phase, we fully integrated the stimulation into the headband to build a real-time closed-loop sleep faster program that Earable can turn off the audio right after automatically detecting the SOL. Here, we introduce a Probability of Being Asleep (PoAs) parameter evaluating the “sleepiness” level by approximating the probability of falling asleep given recently observed data. Suppose the PoAs slope is not sufficiently high (i.e., the user is not falling asleep normally) within the first 20 min, our stimulation mechanism will automatically change the background music through a Content Switching (CW) function. Figure 7f depicts a PoAs example of a participant spending three nights using Earable: Night 1 without and Nights 2–3 with the sleep faster program. Regardless of stimulation conditions, PoAs profiles consist of 3 parts: First, the PoAs rises relatively quickly within several minutes when the participant starts closing her eyes and relaxing. Then, it becomes relatively constant, indicating she is trying to sleep. Finally, it increases and reaches another plateau value, typically>80%, coinciding with the detection of SOL indicated by the solid arrows. Thus, our PoAs metric is a powerful manner of measuring users’ sleep progress. Upon introducing the closed-loop stimulation on Days 2–3, we observe that PoAs profiles can reach the final plateau value faster than without stimulation. In this figure, the participant’s SOLs were 35, 25, and 25.5 min on Days 1–3, respectively, suggesting the effectiveness of our proposed program.

To prove such stimulation effectiveness is statistically significant, we repeated the measurement on different participants. Figure 7g,h illustrate the results in nap and full-night sessions using the Earable headband solely. Specifically, Fig. 7g shows that Earable successfully reduces the SOL from 36.6 ± 10.0 min (Day 1, no stimulation) to 16.9 ± 12.0 min (Day 2, closed-loop stimulation) on 57 participants during naps. The full-night home testing protocol (Fig. 7h) further shows that Earable successfully reduces the SOL from 41.3 ± 15.8 min (Day 1, no stimulation) to 22.3 ± 19.1 min and 26.8 ± 21.7 min (Days 2–3, closed-loop stimulation) over 18 participants.

### User experience survey

We surveyed participants’ experience with Earable to determine the gap in our research and real-life expectations. Particularly, our questionnaire asks about their perspective on Earable’s convenience, comfort, ease of use, and satisfaction with acoustic stimulation. This information provides us insights into system improvement in the future.

**Figure 8:**
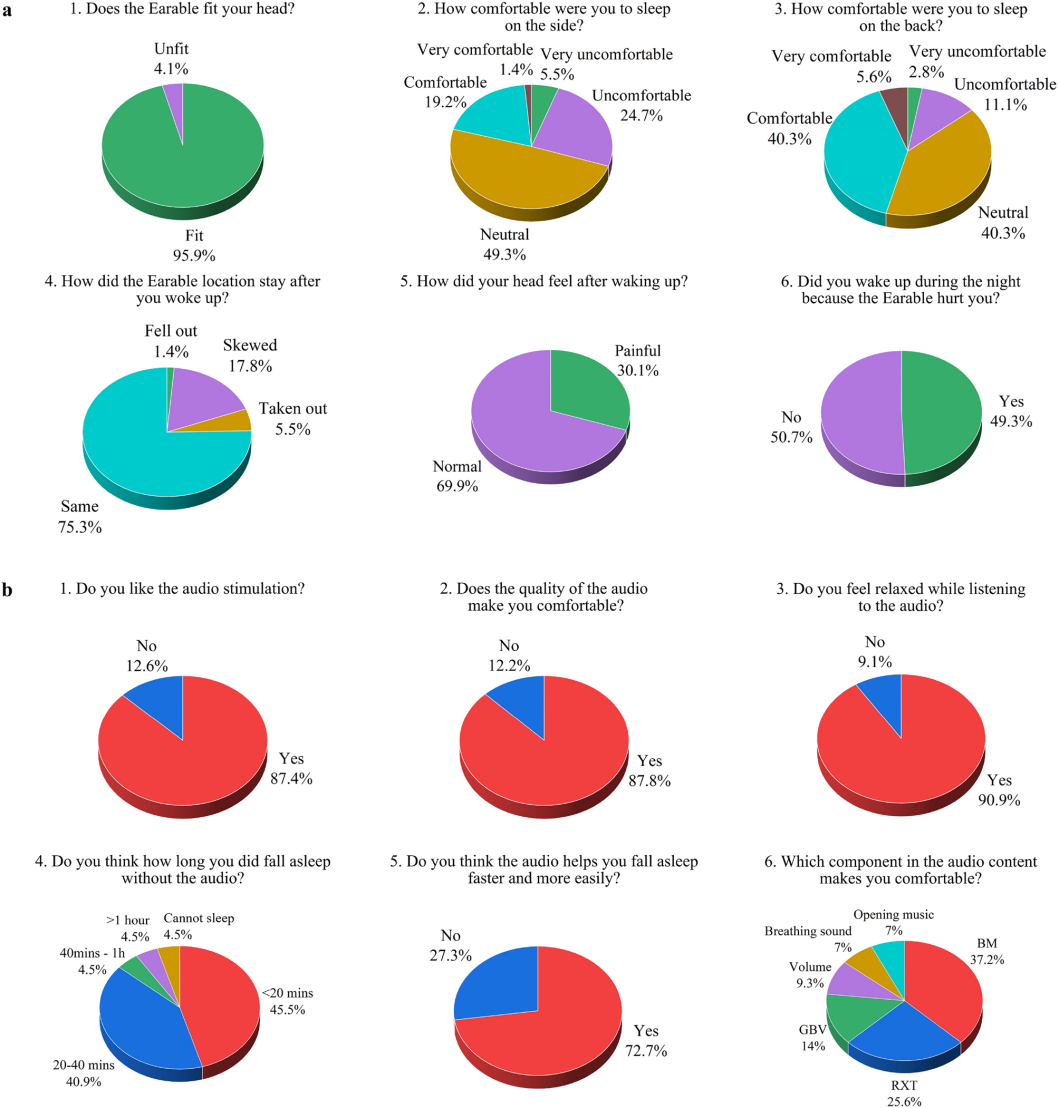
User experience questionnaire results. (**a**) The perspective of convenience, comfort level, and ease of use. (**b**) The perspective of stimulation satisfaction.

Figure 8 shows our questions and participant feedback statistics. In Fig. 8a, more than 95% reported Earable fitted well with their heads. For their first-time usage, more than 69% and 86% positively feel either neutral or comfortable while lying down on their sides and back, respectively, with Earable. Additionally, the headband stayed in place in more than 75% of full-night studies with their free movements. Among nearly half of them waking up during the night, about 69% felt the headband did not hurt their heads. We believe using the device repetitively will make people more comfortable and have a minimum painful effect as they can adjust the band tightness more appropriately.

In Fig. 8b, we asked about their experience with our auditory stimuli. Specifically, more than 87% felt comfortable and relaxed while listening to the audio. Surprisingly, when starting their study, more than 45% imprecisely self-evaluated their duration of falling asleep as < 20 min. Until the end, our audio tracks could help more than 72% fall asleep quickly. Finally, as our audio combines different components, we asked about their favorite components. As expected, the three key components, including guided breathing voice (GBV), relaxation therapy voice (RTV), and background music (BM), contribute significantly to the efficacy of the stimulation as it can lull them into sleep smoothly. Additionally, an appropriate volume is a decent factor contributing to the success of our fast sleep program.

## Discussion

In this work, we proposed a novel head-worn sleep-aid system achieving high-fidelity signal recording, accurate sleep monitoring, and effective stimulation for fast sleep falling in a large-scale study. Foremost, Earable can capture sufficiently informative signals using optimally lightweight hardware. Therefore, compared to PSG with several drawbacks, Earable is more user-friendly for multi-purpose use over days.

Secondly, Earable is powerful for reliable sleep staging with an accuracy comparable to the ones scored by sleep technicians using PSG data. Prior to our research, there were non-head-worn^22–24,29,30^ and contactless sensory systems, especially bed-embedded ones^31–41^, available for sleep monitoring. Among these, a ballistocardiogram bed sensor was developed with an average accuracy of 90.80%^35^. Nevertheless, the sensor was assessed with only five patients and no reported runtime as proof of practical real-time deployment. Additionally, although these are comfortable in use, without the ability to capture brain signals, they cannot precisely analyze sleep stages^48^. Consequently, they are unlikely to deliver on-time stimulation as ours can. Those bed-embedded devices, furthermore, lack the mobility feature. Thus, by leveraging electrophysiological signals, clinically used for sleep diagnosis for years, to primarily guide the sleep staging process and smartly switching to PPG and acceleration data to estimate the sleep stage for noisy physiological sources, Earable can significantly eliminate the number of unscorable epochs. Furthermore, Earable can potentially be integrated with bed-embedded sensors to provide continuous monitoring of vital and brain signals for critical neurological disorders/diseases such as epilepsy or sleep apnea. The continuous tracking feature can provide a better understanding of patients’ condition.

Finally, Earable enables effective auditory stimulation to shorten sleepless participants’ SOL. It is encouraging that more than three-fourths of our subjects fell asleep in a shorter time. Especially our varied baseline design in a couple of the sub-studies gives more confidence that the effectiveness results from the intervention (not a first-night effect or regression to the mean). Here, non-responders to our audio tended to be subjects with a high sleep time on the previous day reported prior to the study, suggesting a ceiling effect with key parameters integrated into the audio. Beyond this direct way of evaluating the efficacy of stimulation based on inter-scorer hypnograms, we further provide sleep variables as alternative macro-metrics statistically computed from the hypnogram to compare sleep characteristics with and without the stimulation in Supplementary Tables ST 4 and ST 5.

However, we also note various limitations to this work. First, we were unable to measure oxygen saturation due to the subtly unstable placement of the PPG sensor on the forehead. Further improvement in the design should solve this issue to enrich the amount of data. Secondly, it would be ideal to increase the auditory intervention’s randomization over more nights. Finally, the sample was somewhat homogeneous in age, about two-thirds of our subjects in their 20s and 30s, and national origin. A larger sample of more diverse sleepers in age and nations would have provided more generalizability to the population. Hence, we should further improve study protocols and broaden the subjects’ demographics.

Overall, all results indicate that the Earable headband holds great potential for advanced sleep care monitoring and improvement at home. This opens exciting possibilities for several exploratory studies in both clinical diagnostics and neuroscience research, such as those combining simultaneous electrophysiology and vital monitoring with various non-invasive brain stimulations to boost the flexibility and effective aiding of systems for sleep and other mental health issues.

## Methods

### Study protocols

We conducted two separate study protocols to prove the accuracy of our sleep staging model and the effectiveness of our auditory stimulation. Both were approved by the Institutional Review Board (IRB) of the Vietnam Society of Sleep Medicine in Protocol No. 01.10.2020-HDDD/HYHGNVN and conducted in accordance with the relevant guidelines and regulations and ethical standards of the 2013 Declaration of Helsinki. Also, informed consent was obtained from all subjects and/or their legal guardian(s). Specifically, we recruited a total of 377 participants (248 females, 129 males) regardless of gender or ethnicity from the local community through advertisement flyers. To confirm eligibility for the studies, volunteers first completed an online questionnaire of a detailed demographic, medical, health, sleep, and lifestyle. Next, we ruled out those with medical conditions, including sleep disorders (except the difficulty of falling asleep) and severe cardiac, neurological, or psychiatric comorbidity. Additionally, none of them had participated in any night-shift work or had traveled across more than one-time zone within a month. We then randomly assigned the eligible participants to either of the studies. They later gave written consent before attending their experimental session. After the sleep session, the technician removed all devices, and participants were debriefed and completed another questionnaire to identify any adverse events and provide feedback. Finally, all participants received financial compensation commensurate with their study involvement. We now describe the details of the two studies and their relevant protocols chronologically.

#### Study I

The purpose of this study is three folds: (1) to assess the reliability of recording electrophysiological and vital signals of Earable’s sensors, (2) to develop the real-time sleep scoring model, and (3) to evaluate its accuracy. One hundred fifty-five (155) healthy participants (84 females, 71 males) aged between 19 and 33, with an average BMI of 21.58 ± 3.31, were recruited. Each participant provided one night of data. Each night, a sleep technician sets up both PSG and the Earable to undergo an overnight sleep study at our sleep lab. The final analysis data set consisted of 155 full-night records. The PSG and Earable data recordings were synchronized a posteriori, so the records were well-aligned.

#### Study II

This study aims to objectively assess the efficacy of our proposed acoustic stimulation that could promote fast sleep by successfully reducing the SOL. We designed multiple study phases to investigate the audio stimulation’s influence on SOL, giving the confidence that the efficiency is likely caused by our intervention and not by other reasons. In addition, no first nap or night was considered for the first-night effect because our eligible subjects got familiar with the PSG and Earable in advance. A total of 305 participants (213 females, 92 males) aged between 19 and 55, with an average BMI of 21.07 ± 2.83 and an average SOL of 39.16 ± 11.66 min, were recruited and randomly assigned to two-, three-, four-nap, or three full-night protocols from Phase 1–3 described below. One day before their first study and during the whole experiment, we asked them to refrain from caffeine and alcohol and maintain a regular sleep and wake pattern, with a daily nocturnal sleep of 7 h.

- Phase 1: we utilized daytime naps to run our studies with the data collected using the PSG. We played the audio through speakers integrated into our headband. During the first 20 min, the participant watched a short video while the technician hooked up all the required electrodes to the participant’s head. All lights were on and windows were open to keep the participant awake. After finishing the setup, we switched off the lights and closed the windows. Then, the participant began the sleep session. After 50 min of sleeping, the participant was woken up, and the study ended.
- Phase 2: we ran the nap studies using only the Earable headband for both stimuli playing and data collection. The participants went through a similar process described in Phase 1 at our sleep lab.
- Phase 3: we evaluated the stimulation efficacy through three full-night sleep studies using only the Earable headband. To mimic the real-world experience for the participants, we gave them the headband to use at their homes and monitored their SOL with and without stimulation from afar. The final analysis data set consisted of 674 nap and 54 full-night records.

### Ground truth sleep data collection and labeling

We performed ground-truth collection using either an Alice 6 LDxS Diagnostic sleep system (Philips, USA)^49^ with the following EEG derivations: F3/A2, F4/A1, C3/A2, C4/A1, O1/A2, O2/A1, LOC/A2, and ROC/A1 at 200Hz sampling rate with a 0.3–35Hz bandpass filter. In addition, the PSG performed bilateral EOG and bilateral chin EMG recordings. It also monitored airflow, thoracic movements, and fingertip oxygen saturation.

For ground-truth labelling, inter-technician contradictions are relatively common when comparing the scoring results from two or more technicians for the same sleep study due to their subjective interpretation of a data section^50^. Consequently, building a set of ground truth labels based on one technician’s scoring can bias machine learning models to learn scoring patterns specific to this technician. Therefore, we constructed a consensus hypnogram for each sleep study using the scoring hypnograms from at least three trained sleep technicians to encourage the model to learn generalizable features. In this approach, the ground truth sleep stage for each epoch is determined to be the maximally voted stage for that epoch by the set of technicians. When a tie exists (i.e., all technicians score the same epoch differently), the epoch is scored according to the technician with the most frequent inter-technician agreement for this study^51^.

### Earable sleep scoring algorithm

Traditionally, according to the AASM scoring manual^45^, technicians partition a sleep study into epochs and assign each to one of five sleep stages, including W, N1 (non-REM 1), N2 (non-REM 2), N3 (non-REM 3 or deep sleep), and REM. Thus, the acquired data is processed and scored after the sleep session, allowing the technician to reference both historical and future data when scoring a given epoch for a more accurate interpretation of the patient’s sleep architecture. On the other hand, we develop the Earable algorithm in the manner of epoch-by-epoch real-time sleep scoring to serve the real-time closed-loop stimulation. Furthermore, in contrast to the 5-stage scoring methodology suggested by the AASM, our sleep scoring algorithm performs 4-stage scoring, clustering N1 and N2 into a higher-level abstraction, light sleep. Motivating this sleep stage aggregation is the low inter-technician agreement when scoring the stages N1 and N2 and the similar biological phenomena observed in these lighter stages of sleep. The Earable sleep staging algorithm is composed of 5 primary sub-modules:

1. A *Channel Selection* module (Supplementary Mod. SM 1), which identifies which data channels of the device contain signals with high enough fidelity for sleep staging
2. A *Dynamic Re-referencing* module (Supplementary Mod. SM 2), which adjusts the default channel referencing scheme depending on the channels inferred to be of good signal quality from the Channel Selection module
3. A *Data Preprocessing and Feature Extraction* module (Supplementary Mod. SM 3), which cleans and summarizes epoch data through the results of digital signal processing and statistical analyses
4. A *Primary Machine Learning* (PML) model (Supplementary Mod. SM 4), which infers a probability distribution of sleep stages using the features computed from EEG, EOG, and EMG signals processed in the former module, and
5. A *Secondary Machine Learning* (SML) model (Supplementary Mod. SM 5), designed to estimate a probability distribution of sleep stages using the features computed from PPG and IMU sensors, provided that electrophysiological signal data has been unstable from all electrode channels for a sufficient amount of time

Deploying this whole algorithm on an iPhone 12 running iOS 16.6 as the host device, we achieved an average runtime of 1.0734 ± 0.0191 seconds while inferring the sleep stage of one 30-second data epoch. In other words, this resulting performance conclusively demonstrates the Earable sleep scoring algorithm’s ability to trigger the sleep stimulation module in real-time.

### Real-time closed-loop fast sleep acoustic stimulation

For effective stimulation, we first distinctively custom-build the audio files consisting of a guided breathing voice (GBV), a relaxation therapy voice (RTV), and background music (BM). The audio volume for each voice is faded out at each appropriate timeline, as indicated in Fig. 7b, to produce the best sleep promotion effect. The GBV is to help users breathe at appropriate paces, the RTV is to help users relax, and the BM is to help users feel relaxed and hence fall asleep easier. As illustrated in Fig. 7b, at t0, we turn on the GBV to help regulate the user’s breathing at a sleep pace while slowly turning on the BM. From t1 to t2, we apply the RTV music to help the user relax and fall asleep. From t2 onward, only the BM remains and will slowly fade out. Next, we fully integrate the custom-built audio stimuli into the headband to develop a real-time actuating sleep faster program. Therefore, Earable can turn off the audio right upon the SOL detection. Leveraging the PoAs parameter introduced in the Evaluation, our stimulation mechanism will automatically change the background music through two dedicated sub-modules.

1. *Automatic content switching* (ACS) module
  Users may respond differently to various audio content sources when trying to fall asleep. While some may find the sounds of rainfall, for example, soothing and sleep-inducing, others may find it distracting. Hence, we develop an automatic content switching (ACS) algorithm that changes the background music in the audio stimulation in response to the real-time sleepiness trend of a given user. This algorithm helps users fall asleep smoothly, without interruption or delay caused by audio stimulation that feels disruptive to falling asleep. As the user falls asleep, their level of sleepiness is continuously inferred every epoch via the PoAs value. During the sleep onset process, if we observe a negative sleepiness trend occurring with the current background music source, this music content will automatically change to guide the user to sleep better.
2. *Audio content recommendation* (ACR) module
  When it is time to select a new source of audio to play for the user (including when the user starts a sleep session, after the current audio finished playing, or when prompted by the ACS algorithm) historical data from the user can be leveraged to select audio content that will optimally encourage sleep induction. The audio content recommendation (ACR) algorithm instantiates the reinforcement learning Multi-Armed Bandit problem^52–56^. A content recommendation agent attempts to choose audio content for the stimulation that will maximize the subject’s PoAs rate of change (suggesting a maximal rate of increase in the subject’s sleepiness level). Audio content preferences for each user are updated by observing rewards from the previous sleep session, where the reward for a given audio content is computed as the user’s PoAs rate of change while that content was being played. The value of a given audio content is represented by a normal distribution, whose parameters are updated after each reward observation for that content. These distributions are specific to each user, as users will naturally have different audio preferences. Initially, without prior knowledge, all audio content has a value distribution with significant variance, as the user’s preference is unknown. Over time, as rewards are observed for a given audio content, the variance of the content’s value distribution decreases as the preference model distribution becomes closer to the true expected reward for that content. The Thompson sampling algorithm^57,58^ is employed to update the preference model distribution parameters over time. When the content recommendation is needed, the value distribution of each audio content (excluding the content that was most recently used) is sampled, and the content yielding the highest value sample is selected. This algorithm allows for custom user preferences without extensive prior user data. New audio content sources may be released at any time without altering other content distribution parameters, as a new value distribution may be instantiated for this content.

### Statistical information

Unless otherwise noted, we performed all statistical analyses comparing mean values between experimental conditions using one-way ANOVA tests with a significance value of 0.05. Upon rejection of the null hypothesis when comparing mean values between more than two groups (i.e., when the resulting p-values were less than 0.05), Tukey’s post-hoc tests were performed among groups, pairwise, and adjusted p-values were reported.

### Supplementary Information

Supplementary Information.

## Data Availability

All data associated with this study are available in the main text or the supplementary materials. Additional sleep dataset generated during the studies is available in the IRB repository and may be requested from A.N. (anh.nguyen@umontana.edu).

## References

[1] Chattu VK (2018). The global problem of insufficient sleep and its serious public health implications. Healthcare.

[2] Altevogt, B. M. *et al.**Sleep Disorders and Sleep Deprivation: An Unmet Public Health Problem* (National Academies Press, 2006). 20669438

[3] Gangwisch JE (2014). Daytime sleepiness and risk of coronary heart disease and stroke: Results from the nurses’ health study. II. Sleep Med..

[4] Freeman D, Sheaves B, Waite F, Harvey AG, Harrison PJ (2020). Sleep disturbance and psychiatric disorders. Lancet Psychiatry.

[5] Angarita GA, Emadi N, Hodges S, Morgan PT (2016). Sleep abnormalities associated with alcohol, cannabis, cocaine, and opiate use: A comprehensive review. Addict. Sci. Clin. Pract..

[6] Fielding JE, Teutsch S, Koh H (2012). Health reform and healthy people initiative. Am. J. Public Health.

[7] St-Onge M-P (2016). Sleep duration and quality: Impact on lifestyle behaviors and cardiometabolic health: A scientific statement from the american heart association. Circulation.

[8] Therapeutics, P. *SILENOR*. https://www.silenor.com/ (2019).

[9] Inc., M. C. *BELSOMRA*s. https://www.belsomra.com/ (2019).

[10] Remrise. *Remrise*. https://remrise.com/ (2019).

[11] Gooneratne NS (2008). Complementary and alternative medicine for sleep disturbances in older adults. Clin. Geriatr. Med..

[12] Ramakrishnan K (2007). Treatment options for insomnia. South Afr. Fam. Pract..

[13] Pagel J, Parnes BL (2001). Medications for the treatment of sleep disorders: An overview. Prim. Care Companion J. Clin. Psychiatry.

[14] Alomar MJ (2014). Factors affecting the development of adverse drug reactions. Saudi Pharm. J..

[15] Ye Y-Y (2015). Internet-based cognitive behavioral therapy for insomnia (icbt-i) improves comorbid anxiety and depression-a meta-analysis of randomized controlled trials. PLoS ONE.

[16] Ye Y-Y (2016). Internet-based cognitive-behavioural therapy for insomnia (icbt-i): A meta-analysis of randomised controlled trials. BMJ Open.

[17] Cheng SK, Dizon J (2012). Computerised cognitive behavioural therapy for insomnia: A systematic review and meta-analysis. Psychother. Psychosom..

[18] Kosmyna N, Maes P (2019). AttentivU: An EEG-based closed-loop biofeedback system for real-time monitoring and improvement of engagement for personalized learning. Sensors.

[19] Horowitz AH, Cunningham TJ, Maes P, Stickgold R (2020). Dormio: A targeted dream incubation device. Conscious. Cognit..

[20] Lee JH (2020). 3D printed, customizable, and multifunctional smart electronic eyeglasses for wearable healthcare systems and human-machine interfaces. ACS Appl. Mater. Interfaces.

[21] Kushida CA (2005). Practice parameters for the indications for polysomnography and related procedures: An update for 2005. Sleep.

[22] Fitbit. *Fitbit*. https://www.fitbit.com/home (2021).

[23] Jawbone. *Jawbone UP*. https://www.jawbone.com/ (2021).

[24] Withings. *Steel hr sport*. https://www.withings.com/us/en/steel-hr-sport (2021).

[25] Dreem. *Dreem 2*. https://dreem.com/en (2021).

[26] Philips. *Smartsleep*. https://www.usa.philips.com/c-e/smartsleep.html (2021).

[27] Sleep Shepherd. *Sleep Shepherd*. https://sleepshepherd.com/ (2021).

[28] Rostaminia S, Homayounfar SZ, Kiaghadi A, Andrew T, Ganesan D (2022). Phymask: Robust sensing of brain activity and physiological signals during sleep with an all-textile eye mask. ACM Trans. Comput. Healthc..

[29] Alqurashi YD (2018). A novel in-ear sensor to determine sleep latency during the multiple sleep latency test in healthy adults with and without sleep restriction. Nat. Sci. Sleep.

[30] Mikkelsen KB (2019). Accurate whole-night sleep monitoring with dry-contact ear-EEG. Sci. Rep..

[31] Schütz N (2021). Contactless sleep monitoring for early detection of health deteriorations in community-dwelling older adults: Exploratory study. JMIR Mhealth Uhealth.

[32] Zhao, M., Yue, S., Katabi, D., Jaakkola, T. S. & Bianchi, M. T. Learning sleep stages from radio signals: A conditional adversarial architecture. In *International Conference on Machine Learning* 4100–4109 (PMLR, 2017).

[33] Peng M, Ding Z, Wang L, Cheng X (2019). Detection of sleep biosignals using an intelligent mattress based on piezoelectric ceramic sensors. Sensors.

[34] Siyahjani F, Garcia Molina G, Barr S, Mushtaq F (2022). Performance evaluation of a smart bed technology against polysomnography. Sensors.

[35] Gargees, R., Keller, J. M., Popescu, M. & Skubic, M. Non-invasive classification of sleep stages with a hydraulic bed sensor using deep learning. In *How AI Impacts Urban Living and Public Health: 17th International Conference, ICOST 2019, New York City, NY, USA, October 14-16, 2019, Proceedings 17* 73–82 (Springer, 2019).

[36] Zhang, L. *et al.* Sleep stages classification by cw doppler radar using bagged trees algorithm. In *2017 IEEE Radar Conference (RadarConf)* 0788–0791 (IEEE, 2017).

[37] Yi, R., Enayati, M., Keller, J. M., Popescu, M. & Skubic, M. Non-invasive in-home sleep stage classification using a ballistocardiography bed sensor. In *2019 IEEE EMBS International Conference on Biomedical & Health Informatics (BHI)* 1–4 (IEEE, 2019).

[38] Zhang F (2019). Smars: Sleep monitoring via ambient radio signals. IEEE Trans. Mobile Comput..

[39] Sleep Cycle. *Sleep Cycle App*. https://www.sleepcycle.com/ (2019).

[40] SleepScore. *SleepScore App*. https://www.sleepscore.com/ (2019).

[41] Nálevka), U. P. *Sleep As Android*. https://play.google.com/store/apps/details?id=com.urbandroid.sleep&hl=en_US (2019).

[42] Xu J, Mitra S, Van Hoof C, Yazicioglu RF, Makinwa KA (2017). Active electrodes for wearable EEG acquisition: Review and electronics design methodology. IEEE Rev. Biomed. Eng..

[43] Patel, A. K., Reddy, V. & Araujo, J. F. Physiology, sleep stages. In *StatPearls [Internet]* (StatPearls Publishing, 2022). 30252388

[44] Leach S, Chung K-Y, Tüshaus L, Huber R, Karlen W (2020). A protocol for comparing dry and wet EEG electrodes during sleep. Front. Neurosci..

[45] American Academy of Sleep Medicine™. AASM. https://aasm.org/ (2021).

[46] Cohen J (1960). A coefficient of agreement for nominal scales. Educ. Psychol. Meas..

[47] Wang Y, Loparo KA, Kelly MR, Kaplan RF (2015). Evaluation of an automated single-channel sleep staging algorithm. Nat. Sci. Sleep.

[48] Hussain Z, Sheng QZ, Zhang WE, Ortiz J, Pouriyeh S (2022). Non-invasive techniques for monitoring different aspects of sleep: A comprehensive review. ACM Trans. Comput. Healthc..

[49] Philips. *Alice 6 LDxS PSG Sleep System*. https://tinyurl.com/mt6bebc (2021).

[50] Rosenberg RS, Van Hout S (2013). The American academy of sleep medicine inter-scorer reliability program: Sleep stage scoring. J. Clin. Sleep Med..

[51] Arnal PJ (2020). The dreem headband compared to polysomnography for electroencephalographic signal acquisition and sleep staging. Sleep.

[52] Berry, D. A. & Fristedt, B. *Bandit Problems: Sequential Allocation of Experiments (Monographs on Statistics and Applied Probability)* **5**, 7 (Chapman and Hall, 1985).

[53] Cesa-Bianchi, N. & Lugosi, G. *Prediction, Learning, and Games* (Cambridge University Press, 2006).

[54] Gittins, J., Glazebrook, K. & Weber, R. *Multi-armed Bandit Allocation Indices* (Wiley, 2011).

[55] Bubeck S, Cesa-Bianchi N (2012). Regret analysis of stochastic and nonstochastic multi-armed bandit problems. Found. Trends Mach. Learn..

[56] Slivkins A (2019). Introduction to multi-armed bandits. Found. Trends Mach. Learn..

[57] Russo DJ (2018). A tutorial on thompson sampling. Found. Trends Mach. Learn..

[58] Thompson WR (1933). On the likelihood that one unknown probability exceeds another in view of the evidence of two samples. Biometrika.

