# Supplementary material for: A comprehensive study on the efficacy of a wearable sleep aid device featuring closed-loop real-time acoustic stimulation
Source: Sci Rep. 2023 Oct 16;13:17515. doi: 10.1038/s41598-023-43975-1 (PMC10579321; doi:10.1038/s41598-023-43975-1)
Supplement: Supplementary file 1 — Supplementary Information. [file 41598_2023_43975_MOESM1_ESM.pdf]

Supplementary Information for A Comprehensive Study on the Efficacy of a Wearable Sleep Aid Device Featuring Closed-Loop Real-Time Acoustic Stimulation

Anh Nguyen[1,*], Galen Pogoncheff[2], Ban Xuan Dong[2], Nam Bui[3], Hoang Truong[4], Nhat Pham[5], Linh Nguyen[2], Hoang Nguyen-Huu[6], Khue Bui-Diem[6], Quan Vu-Tran-Thien[6], Sy Duong-Quy[7,8,9], Sangtae Ha[2,4], and Tam Vu[2,4,10]

[1]University of Montana, Department of Computer Science, Missoula, Montana, 59812, USA
[2]Earable Inc., Boulder, Colorado, 80309, USA
[3]University of Colorado Denver, Department of Electrical Engineering, Denver, Colorado, 80204, USA
[4]University of Colorado Boulder, Department of Computer Science, Boulder, Colorado, 80309, USA
[5]Cardiff University, School of Computer Science and Informatics, Cardiff, CF24 4AG, UK
[6]University of Medicine and Pharmacy at Ho Chi Minh City, Vietnam
[7]Lam Dong Medical College, Da Lat City, Lam Dong Province, Vietnam
[8]Pham Ngoc Thach University of Medicine, Ho Chi Minh City, Vietnam
[9]Hershey Medical Center, Penn State College of Medicine, Hershey, PA, 17033, USA
[10]University of Oxford, Department of Computer Science, Oxford, OX1 3QD, UK
*anh.nguyen@umontana.edu

Supplementary Information

Supplementary Module SM 1: Channel selection

EEG, EOG, and EMG signal qualities highly depend on the contact quality of a given electrode with the user's scalp. A body movement throughout the night may cause displacement of the headband on the head, disrupting the contact of one or more electrodes and therefore causing the signal quality to become unsatisfactory for sleep scoring from these channels. Hence, we develop the Channel Selection algorithm as a lightweight Decision Tree classifier[1] which takes as input summary signal quality features from a single signal channel at the current epoch and outputs a binary result indicating the signal quality of the channel at this epoch (i.e., acceptable for scoring or unacceptable for scoring). This signal quality classification is performed for each of the six default headband channels to establish a set of "scorable" channels. Alternatively, we apply rule-based channel selection sub-modules to establish the fidelity of the three PPG signals for SML model inference. Besides that, an outlier detection method is then applied to reject IMU samples associated with irregular sensor movement and readings. These sub-modules establish the utility of PPG and IMU data at the current epoch for sleep scoring.

Supplementary Module SM 2: Dynamic re-referencing

We perform signal referencing for EEG, EOG, and EMG signals to obtain signals with higher signal-to-noise ratios and fewer artifacts. By default, all channels are referenced to the CMS electrode of the headband. If either of the BE electrodes of the headband is found to have acceptable signal quality (as evaluated by the Channel Selection module), these channels are introduced as new reference channels. Specifically, if the BE_L channel has acceptable signal quality, the right-hand side channels (FH_R, OTE_R, and BE_R) are re-referenced to BE_L (provided that they were deemed scorable by the Channel Selection module). This same re-referencing process is performed analogously for the left-hand side channels (FH_L, OTE_L, and BE_L) if and only if the BE_R channel has been inferred to have acceptable signal quality.

Supplementary Module SM 3: Data pre-processing and feature extraction

After channel selection and dynamic re-referencing, a set of channels is available for sleep staging inference. Each of these channels is pre-processed through signal clipping to limit the amplitude range to [-500, 500] uV and applied by a set of notch filters to remove powerline noise in the raw signal. Filters designed to isolate EEG, EOG, and EMG components from each signal are then applied to obtain surrogate EEG, EOG, and EMG signal data from each channel. A spectrogram is computed

from the EEG signal component, and a 38-dimensional feature vector is computed to summarize time and frequency domain analyses from each channel's EEG, EOG, and EMG components. Two PML model input tensors are finally computed from each channel.

Supplementary Module SM 4: Primary machine learning (PML) EEG/EOG/EMG-based model

Morphological aspects of biosignal data critical to sleep stage inference and interpretation are present in the time, frequency, and time-frequency domains. Hence, in this module, we employ both recurrent and convolutional neural sub-networks in a hybrid input manner to leverage such spatial and time-dependent features. Specifically, the PML model takes inputs as the spectrogram and the 38-dimensional feature vector for each epoch. The spectrogram is then fed into a shallow, 2-layer convolutional neural sub-network. On the other hand, the feature vector, in addition to 7 epochs of historical feature data, is fed to the recurrent neural sub-network to obtain an additional feature mapping. Output vectors from each subnetwork are concatenated to achieve a 928-dimensional latent feature vector which is finally presented to a single-layer dense classification head. Finally, we apply a final softmax layer[2] to the 4-unit output to achieve a probability distribution over the four sleep stage classes (i.e., W, LS, DS, and REM). At each epoch, this spectrogram and feature vectors are computed for each channel with acceptable signal quality. A forward pass through the network is performed using these data representations. If multiple channels have acceptable signal quality, each channel's sleep stage probability distributions will be averaged to obtain an ensembled sleep stage confidence estimate. Finally, the estimated sleep stage for the epoch is the sleep stage associated with the highest confidence estimate.

The PML model architecture is tuned in the k-fold cross-validation[3] and designed to enable an optimal balance of size and efficiency to perform accurate, real-time sleep scoring on a user's mobile device. Specifically, model parameters were learned using a training/validation dataset composed of 106 randomly selected sleep studies, which accounted for 68% of the 155 total sleep studies. In this process, we set k to 11 such that each fold had 96 sleep sessions for training and 10 for validation. It is worth noting that the final fold had only 6 validation sessions due to the total count of training sessions being 106. As averaged over the 11 folds, we achieved an accuracy of $84.08 \pm 1.42\%$. Details on this model evaluation are provided in Supplementary Tab. ST 1. However, a primary challenge in developing algorithms for performing inference based on electrophysiological signals is an inter-user generalization. Many components of the EEG, EOG, and EMG signals vary substantially across people, which makes the development of models that do not overfit the data it was trained on challenging. Furthermore, signal morphology is influenced by the hardware used for data acquisition, reducing the efficacy of simply training a model trained on large data sets acquired using clinical PSG hardware. Consequently, we perform a two-step training process of pre-training and then fine-tuning to mitigate the performance issues that can arise from these challenges.

In the pre-training phase of the PML model training, we train the network using sleep data acquired from clinical-grade PSG devices. It is because the signal hallmarks that sleep technicians use to stage sleep are most visible in this gold-standard data, compared to data acquired from typical wearable devices since the hardware is carefully configured by the technician and is consistently monitored throughout the night and adjusted as needed. By pre-training the PML model on this PSG data from subjects of varying demographics (age, gender, etc.), our model learns signal features critical for scoring and generalization across users. While training, we stochastically optimize the model parameters using the Adam optimizer[4], iteratively minimizing categorical cross-entropy loss. Early stopping was used to terminate pre-training by monitoring classification accuracy on a withheld validation dataset[5].

Naturally, the distribution of spectrogram and feature vector values in the Earable headband data will differ from those in the clinical grade PSG data due to the different hardware configurations. The pre-trained PML model must be tuned following different data distributions. In this tuning process, we freeze the layers of the convolutional subnetwork while updating the layers of the recurrent sub-network and classification head by training on sleep data acquired using the Earable headband. Similar to the pre-training phase, we apply the Adam optimization in this iterative process. Moreover, we use a lowered learning rate and label-smoothing[6] of ground truth consensus labels to stabilize the optimization process in the presence of epochs with noisy data that do not contain data reflective of the ground truth sleep stage.

Supplementary Module SM 5: Secondary machine learning (SML) PPG and IMU-based model

Although infrequent, it is possible for all electrodes to lose stable contact with the user's scalp during sleep. In this case, no scorable channel will be available after channel selection. As a result, no reliable EEG, EOG, and EMG information will be available for scoring. In this case, we employ the SML model, which leverages features computed from the PPG and IMU sensors to infer the current sleep stage. Given that sleep stage transitions are typically apparent at more coarse time granularities from these data sources, as compared to EEG, EOG, and EMG signals, this model estimates the current sleep stage of the user using two epochs (i.e., every minute) until reliable electrophysiological signals become available again.

Analogous to sleep staging with EEG, EOG, and EMG signals, the change in biological processes over time provides valuable information for estimating the current sleep stage. We use a shallow recurrent neural network architecture in this SML model. The input to this model is a set of 24 features computed from PPG and IMU time-series data, including the estimated heart rate, respiratory rate, and movement information. In the development process of the Earable headband, the IMU and

PPG sensors were only available for 27 of the total 155 sleep studies. Hence, we conduct model development, training, and validation on the entire set of 27 sleep sessions to ensure that the model is not data-limited by splitting validation data from the 25 training sessions (except 26 in the last fold). As averaged over the 14 folds, we achieved an accuracy of $60.72 \pm 7.68\%$. Supplementary Tab. ST 3 further provides the detailed results. Our findings suggest that this model's capacity to generalize to unseen data is bottlenecked by the amount of training data, and further improvements to the SML model may be attainable with a larger dataset. Additionally, due to the limitation of data, we train this SML model in a typical, iterative method using the Adam optimizer.

Supplementary Module SM 6: Offline Smoothing with a Rule-based scoring and Hidden Markov Model smoothing

If real-time scoring is not required, for instance, when reviewing a historical hypnogram, epochs that originally were unscorable using both the PML and SML models can be scored by applying rule-based scoring decisions and a Hidden Markov Model (HMM) based smoothing algorithm. In a two-pass process, for each unscored epoch, the stage of the epoch is first estimated using a small set of rules following AASM scoring manual procedures and statistical heuristics. Next, the Viterbi algorithm[7] is performed over the sequence of all inferred sleep stages of the hypnogram to compute the most likely sequence of stages in the hypnogram. If a more likely sequence of sleep stages is found in this algorithm, the hypnogram of the user is adjusted accordingly. This not only enables scoring of originally unscored sleep stages (permitting 100% of epochs to be scored in this test dataset, Fig. 6a), but also helps to remove unlikely sleep stage transitions that were inferred by the PML and SML models when processing abnormal signals. HMM transition and emission probabilities were statistically computed from ground truth training data hypnograms[8–14].

Supplementary Function SF 1: Heart rate estimation from PPG signals

Using a 5-second, non-overlapping window, we segment the signals collected from the three PPG channels (IR, Red, and Green) to estimate a user's heart rate. First, the signal segments of each channel that have high enough fidelity according to the PPG channel selection algorithm are bandpass filtered to isolate the AC components of the signal. However, the channels that do not pass this channel selection step are ignored for the current timestep. Then, the resulting filtered signals are normalized to zero mean and unit variance and aggregated by taking the mean of each filtered and normalized signal segment. Systolic peaks are detected in this resulting signal, and heart rate estimates are computed using the durations between successive systolic peaks. We finally apply moving average smoothing to reduce the occurrence of sudden changes in heart rate estimation due to signal noise.

Supplementary Function SF 2: Respiratory rate estimation from IMU signals

To estimate the respiratory rate of a user every minute, we use a 60-second, non-overlapping window of IMU data. Given the orientation of the IMU in the Earable headband, small movements resulting from inhalation and exhalation are detectable via the Y-axis data of the IMU. After applying a bandpass filter to this time-series data to isolate breathing frequency components, we detect respiratory oscillations via the peaks and troughs of the filtered data. The median duration between successive peaks in this 60-second window is finally computed to estimate the user's respiratory rate at this timestep.

References

1. Breiman, L., Friedman, J., Stone, C. J. & Olshen, R. A. *Classification and regression trees* (CRC press, 1984).
2. Goodfellow, I., Bengio, Y. & Courville, A. 6.2. 2.3 softmax units for multinoulli output distributions. *Deep. learning* **180** (2016).
3. Mosteller, F. & Tukey, J. W. Data analysis, including statistics. *Handb. social psychology* **2**, 80–203 (1968).
4. Kingma, D. P. & Ba, J. Adam: A method for stochastic optimization. *arXiv preprint arXiv:1412.6980* (2014).
5. Yao, Y., Rosasco, L. & Caponnetto, A. On early stopping in gradient descent learning. *Constr. Approx.* **26**, 289–315 (2007).
6. Szegedy, C., Vanhoucke, V., Ioffe, S., Shlens, J. & Wojna, Z. Rethinking the inception architecture for computer vision. In *Proceedings of the IEEE conference on computer vision and pattern recognition*, 2818–2826 (2016).
7. Forney, G. D. The viterbi algorithm. *Proc. IEEE* **61**, 268–278 (1973).
8. Ghimatgar, H., Kazemi, K., Helfroush, M. S. & Aarabi, A. An automatic single-channel eeg-based sleep stage scoring method based on hidden markov model. *J. neuroscience methods* **324**, 108320 (2019).
9. Jiang, D., Lu, Y.-n., Yu, M. & Yuanyuan, W. Robust sleep stage classification with single-channel eeg signals using multimodal decomposition and hmm-based refinement. *Expert. Syst. with Appl.* **121**, 188–203 (2019).

10. Pan, S.-T., Kuo, C.-E., Zeng, J.-H. & Liang, S.-F. A transition-constrained discrete hidden markov model for automatic sleep staging. *Biomed. engineering online* **11**, 1–19 (2012).

11. Doroshenkov, L., Konyshev, V. & Selishchev, S. Classification of human sleep stages based on eeg processing using hidden markov models. *Biomed. Eng.* **41**, 25 (2007).

12. Doroshenkov, L. & Konyshev, V. Usage of hidden markov models for automatic sleep stages classification. In *Russian-Bavarian Conference on Bio-Medical Engineering*, 19 (Citeseer, 2007).

13. Flexer, A., Gruber, G. & Dorffner, G. A reliable probabilistic sleep stager based on a single eeg signal. *Artif. intelligence Medicine* **33**, 199–207 (2005).

14. Flexerand, A., Dorffner, G., Sykacekand, P. & Rezek, I. An automatic, continuous and probabilistic sleep stager based on a hidden markov model. *Appl. Artif. Intell.* **16**, 199–207 (2002).

Supplementary Tables

Supplementary Table ST 1. Average performance details of the Earable 4-stage sleep scoring algorithm using k-fold cross validation with 106 sleep studies (k=11).

	Wake	Light Sleep	Deep Sleep	REM
F1-score	0.8482 ± 0.0283	0.8343 ± 0.0161	0.8768 ± 0.0146	0.7960 ± 0.0281
Precision	0.8591 ± 0.0375	0.8470 ± 0.0234	0.8868 ± 0.0227	0.7516 ± 0.0373
Recall	0.8387 ± 0.0338	0.8224 ± 0.0204	0.8684 ± 0.0319	0.8474 ± 0.0316

Supplementary Table ST 2. Performance details of the Earable 4-Stage sleep scoring algorithm using the 49-study independent test set.

	Wake	Light Sleep	Deep Sleep	REM
F1-score	0.90	0.87	0.91	0.82
Precision	0.91	0.87	0.90	0.81
Recall	0.88	0.87	0.91	0.84

Supplementary Table ST 3. Average performance details of the secondary machine learning (SML) algorithm using k-fold cross validation (k=14).

	Wake	Light Sleep	Deep Sleep	REM
F1-score	0.6408 ± 0.1614	0.4882 ± 0.1381	0.6930 ± 0.0838	0.5515 ± 0.1017
Precision	0.5755 ± 0.1853	0.6816 ± 0.1010	0.6508 ± 0.1176	0.5220 ± 0.1375
Recall	0.7560 ± 0.1442	0.4036 ± 0.1489	0.7815 ± 0.1382	0.6376 ± 0.1644

Supplementary Table ST 4. Sleep variables as macro metrics computed on the hypnogram of PSG data collected in the three nap protocols.

	2-Day Nap Protocol 166 participants (130 females, 36 males) (23±2.72 y/o, 21.27±2.93 BMI)		3-Day Nap Protocol 28 participants (17 females, 11 males) (22 ± 4.08 y/o, 21.14 ± 2.56 BMI)		
	Nap 1 No Stimulation	Nap 2 Stimulation	Nap 1 No Stimulation	Nap 2 Stimulation	Nap 3 Stimulation
SOL (min)	33.27 ± 8.52	14.92 ± 8.65	31.13 ± 6.66	12.57 ± 7.75	14.04 ± 8.97
SE (%)	15.12 ± 17.39	55.27 ± 23.56	25.62 ± 20.34	61.10 ± 25.03	53.96 ± 31.93
LS (min)	6.21 ± 7.07	19.10 ± 9.59	7.96 ± 6.71	15.82 ± 7.77	13.46 ± 8.46
LS (% of TIB)	50.43 ± 45.95	69.85 ± 29.79	40.77 ± 36.86	53.00 ± 24.79	47.37 ± 30.20
DS (min)	1.51 ± 4.36	9.11 ± 10.64	6.86 ± 8.43	15.82 ± 10.63	17.13 ± 13.73
DS (% of TIB)	6.99 ± 18.90	25.52 ± 26.48	27.08 ± 30.31	42.47 ± 23.85	39.59 ± 28.48
REM (min)	0.08 ± 0.83	0.15 ± 0.75	0.00 ± 0.00	0.41 ± 1.25	1.30 ± 3.70
REM (% of TIB)	0.41 ± 3.91	0.41 ±1.96	0.00 ± 0.00	0.97 ± 2.96	2.33 ± 6.15

	4-Day Nap Protocol 36 participants (12 females, 24 males) (22.63 ± 2.57 y/o, 20.86 ± 3.03 BMI)			
	Nap 1 No Stimulation	Nap 2 No Stimulation	Nap 3 Stimulation	Nap 4 Stimulation
SOL (min)	34.63 ± 10.05	34.04 ± 7.20	18.86 ± 10.22	17.69 ± 10.85
SE (%)	12.96 ± 16.67	17.41 ± 17.50	46.45 ± 26.76	53.45 ± 25.16
LS (min)	5.51 ± 7.08	7.33 ± 6.97	18.32 ± 11.27	20.40 ± 10.43
LS (% of TIB)	45.77 ± 46.75	57.85 ± 44.28	69.17 ± 31.26	71.83 ± 28.11
DS (min)	1.81 ± 5.01	2.08 ± 4.49	6.40 ± 8.20	7.93 ± 8.31
DS (% of TIB)	7.01 ± 18.84	8.81 ± 18.06	19.04 ± 21.01	22.34 ± 22.62
REM (min)	0.00 ± 0.00	0.00 ± 0.00	0.18 ± 0.80	0.14 ± 0.82
REM (% of TIB)	0.00 ± 0.00	0.00 ± 0.00	0.68 ± 3.25	0.28 ± 1.63

Supplementary Table ST 5. Sleep variables as macro metrics computed on the hypnogram of Earable data collected in the nap and full-night study protocols.

	2-Day Nap Protocol 57 participants (44 females, 13 males) (23±2.98 y/o, 20.69±2.67 BMI)		3-Full Night Protocol 18 participants (10 females, 8 males) (25.36 ± 7.01 y/o, 20.80 ± 2.47 BMI)		
	Nap 1 No Stimulation	Nap 2 Stimulation	Night 1 No Stimulation	Night 2 Stimulation	Night 3 Stimulation
SOL (min)	31.20 ± 8.77	16.99 ± 10.20	43.53 ± 20.64	29.00 ± 21.80	30.44 ± 23.21
SE (%)	29.34 ± 33.73	71.93 ± 45.62	69.70 ± 23.06	80.05 ± 21.45	73.59 ± 28.40
LS (min)	7.23 ± 8.39	14.88 ± 10.46	128.67 ± 60.43	148.75 ± 43.73	127.53 ± 66.19
LS (% of TIB)	51.06 ± 44.15	63.88 ± 35.63	52.12 ± 18.51	47.47 ± 14.50	45.37 ± 20.33
DS (min)	2.30 ± 4.66	7.19 ± 9.08	80.92 ± 41.98	101.53 ± 40.87	78.92 ± 43.09
DS (% of TIB)	10.34 ± 19.43	22.08 ± 26.13	30.30 ± 14.87	31.53 ± 10.68	29.57 ± 15.99
REM (min)	0.00 ± 0.00	0.00 ± 0.00	35.31 ± 28.79	50.14 ± 26.89	42.39 ± 36.02
REM (% of TIB)	0.00 ± 0.00	0.00 ± 0.00	12.05 ± 9.17	15.44 ± 7.10	13.95 ± 9.34
